# Effect of vapor pressure deficit on growth and yield of pearl millet germplasm originating from semi-arid, semi-humid and humid regions

**DOI:** 10.3389/fpls.2024.1465686

**Published:** 2024-12-23

**Authors:** Hadizatou Garba, Falalou Hamidou, Harou Abdou, James Burridge, Vincent Vadez

**Affiliations:** ^1^ Department of Biology, Faculty of Science, University Abdou Moumouni of Niamey/Niger (UAM), Niamey, Niger; ^2^ International Crops Research Institute for the Semi-Arid Tropics (ICRISAT) Sahelian Centre, Niamey, Niger; ^3^ Department of Biology, Faculty of Science, University André Salifou of Zinder/Niger (UAS), Zinder, Niger; ^4^ University of Montpellier, Institute de Recherche pour le Développement (IRD), Diversité, adaptation et développement des plantes (DIADE) Research Unit, Montpellier, France; ^5^ Centre d’Étude Régional pour l’Amélioration de l’Adaptation à la Sécheresse (CERAAS), Thiès, Senegal

**Keywords:** germplasm, vapour pressure deficit, climate change, breeding, atmospheric drought

## Abstract

**Introduction:**

The increase in vapor pressure deficit (VPD) is among the expected change in futur climate, and understanding its effect on crop growth is of much significance for breeeding programs. Three groups (G1,G2 and G3) of pearl millet germplasm, originating from regions with different rainfall intensities, were grown in the field during period of high and low VPDs. The groups G1,G2 and G3 were respectively from Guinean (rainfall above 1000 mm), Soudanian (rainfall between 600 mm and 900 mm), and Sahelian zones (rainfall between 600 and 300 mm) of Africa. The objective was to assess if there was any growth response difference among the germplasm groups.

**Method:**

Four trials were conducted, two in the dry season of 2019 (Ds19) and 2020 (Ds20) with avarage VPDs of 3.62 kPa and 2.92 kPa, respectively, and two in the rainy season of 2019 (Rs19) and 2020 (Rs20) with avaerage VPDs of 1.14 kPa and 0.61 kPa, respectively.

**Results:**

In order to avoid possible confounding effects of radiation on millet growth and yield, data were normalized by the quantity of light received during each season. After this normalization, leaf area and grain yield decreased in the highest-VPD seasons whereas tiller number decreased only in Ds19 (one high VPD season). The comparison of the three germplasm groups indicates that G3 the germplasm group from Sahelian regions showed greater tolerance to high VPD than G1 and G2.

**Discussion:**

Germplasm from the G3 group could be a good material for developing tolerant germplasm to future climate that is bound to have high VPD.

## Introduction

Pearl millet [*Pennisetum glaucum* (L.) R. Br] is one of the main staple food for millions of people in Africa and India. Although more tolerant than other crops, it is also vulnerable to climate change (Rhoné et al., 2020). Throughout most of its production zone, pearl millet is grown as a rainfed crop, with no additional irrigation (Vigouroux et al., 2011). Therefore, the yields are very dependent on climate and its variation (Spencer and Sivakumar, 1986). In West Africa particularly, it is grown in three bioclimatic zones. Two of these bioclimatic zones suffer from low and erratic rainfall (Kumarl and Rao Appa, 1986). In addition to the low and erratic rainfall that limits grain yield (FAO and ICRISAT, 1996), the vapor pressure deficit (VPD) is expected to increase in future climates (Vadez et al., 2012a; Brun et al., 2022; Grossiord et al., 2020) and already prevails in semiarid tropical climates because of the high temperatures (Hamidou et al., 2013).

The VPD is known as one of the main driving forces of transpiration (Will et al., 2013) because it is directly related to stomatal function of plants (Lihavainen et al., 2016). The effect of high VPD on plants is not necessarily related to the soil water status, so that even if soil has abundant water to support plant transpiration, plants facing high VPD can be exposed to highly negative and stressful leaf water potential that can be assimilated to an atmospheric drought (Qing et al., 2022; Koehler et al., 2023). The issue with high-VPD conditions is that CO_2_ assimilation under these conditions is extremely water costly and decreases transpiration efficiency (Sinclair et al., 2005; Vadez et al., 2014). High VPD is also known to reduce the leaf expansion rate, which eventually reduces the leaf area and then decreases the plant’s capacity to intercept solar radiation (Reymond et al., 2003). In relation to this, high-VPD conditions, independently from soil water status, are also reported to reduce plant growth (Leuschner, 2002; Yuan et al., 2019), negatively impacting yield (Hsiao, 2019) and can even induce plant mortality (Will et al., 2013) because of hydraulic failure (McDowell et al., 2008).

So as future climate is bound to face higher VPD conditions, crops therefore need to be prepared to it. First, crops might need to draw more water from the soil to match the water requirement to the water demand. This response could lead to a rapid depletion of soil moisture, thereby increasing the risk of experiencing drought stress faster (Vadez et al., 2024), particularly if high-VPD conditions are combined with reduced precipitation (Duan et al., 2014; Will et al., 2013). On the other hand, crops’ growth and potential yield could be reduced, because of an impaired canopy development, even if soil water is not limiting. Genotypes adapted to these conditions must therefore be searched, in particular those that would be able to sustain plant growth even under high VPD, and be directed as donors into breeding programs aiming at developing climate-ready and resilient cultivars for the future. So in this study, we targeted germplasm grown in Africa (although we included one germplasm from India which represents the second center of pearl millet diversity), originating from zones varying for their rainfall. Besides being very vulnerable to climate change, this region is a large center of diversity of pearl millet (Brunken et al., 1977; Haussmann et al., 2006; Pucher et al., 2015). No study has attempted so far to test possible effects of high-VPD conditions on the growth of pearl millet germplasm coming from contrasting rainfall regions.

The objective of this study was then to assess whether there was a genetic variation among germplasm that are endemic to dry and humid areas, in their growth and agronomic response to high-VPD conditions. Specifically, in this study we assessed growth and yield of three germplasm groups of pearl millet (G1, G2, G3), coming respectively from humid, semi-humid, and dry areas of Africa and India, when affected by VPD. We hypothesized that (i) high VPD would reduce growth and yield of pearl millet germplasm, this effect possibly depending on their origin, and (ii) germplasm from arid zones would be the most tolerant to high VPD, assuming that this germplasm has been recurrently exposed to high-VPD conditions and has then evolved to be adapted to these adverse conditions. In this work, we test in particular if germplasm originating from drier area would be able to sustain leaf area development for radiation interception despite known negative effects of high VPD on leaf area development.

## Materials and methods

The three germplasm groups of pearl millet selected for this study come from geographical gradients of rainfall in Africa and India. A total of 29 genotypes out of the 30 are native to Africa according to this distribution: 10 genotypes came from the Guinean zones (above 1,000 mm per year), 10 other genotypes came from Soudanian zones (600 mm–900 mm per year), and the remaining 9 are from the Sahelian zones (300 mm–600 mm per year) (Boulvert, 1992; Kumarl and Rao Appa, 1986). Only one genotype is native to the arid regions of India and was allocated to the Sahelian group. The genotypes were selected in the Pearl Millet inbred Germplasm Association Panel (PMiGAP). The seeds were obtained from the International Crops Research Institute for the Semi-Arid Tropics (ICRISAT), Sahelian Center, Sadoré, Niger.

### Timing of trials and experimental conditions

Experiments were conducted in the field at the ICRISAT research station in Sadoré (13°N, 2°E). Four experiments were conducted: two in the dry seasons of 2019 and 2020 (Ds19, Ds20) and two in the rainy seasons of 2019 and 2020 (Rs19, Rs20). These periods were chosen to expose the germplasm groups to high VPD in the dry season (high temperature, low relative humidity) and low VPD in the rainy season (low temperature and high relative humidity). Trials were planted in 2019 on end March and end July and were repeated at approximately the same dates in 2020. The experimental layout was a completely randomized block design with four replications for each genotype. Plot was 1 m × 3 m with a plant density of 15 plants/plot.

### Growing conditions and measurements

Experimentation during the dry seasons of 2019 (Ds19) and 2020 (Ds20) was conducted from March to June in both years. All plants were regularly irrigated to avoid any water stress until harvest. Rainy season trials in 2019 (Rs19) and 2020 (Rs20) were conducted under the rainfall regime from July to October. When the rain was interrupted, an irrigation was applied if necessary to avoid soil water deficit for the crop. Temperature and relative humidity (RH) were recorded with a data logger (Tiny tag) installed in the field. Recorded temperature and RH data were used to calculate the VPD with the following formula:


$$\text{VPD}=((100-\text{RH}\%)/100{)}^{*}\left(0.6569^{*}\text{EXP}\left(0.0619^{*}\text{temperature}\right)\right)$$

The photoperiods and radiation data, which have great impact on pearl millet phenology, were obtained from NASA.

To score the VPD effect on growth of the three germplasm groups, agronomic characteristics were assessed during the experiment and at maturity. The stem height was measured and the number of tillers was counted until the head emergence. On the date of female flower appearance, three representative plants were harvested in each plot to measure the leaf area using a leaf area meter (LI-3100C area meter, LI-COR BIOSCIENCES, USA). The stems and leaves of harvested plants were oven dried for 72 h at 70°C to determine the dry biomass. The grain yield was estimated at maturity on three plants representative of each genotype chosen in the center of the plot.

### Data analysis

The different seasons received different amounts of radiation, in part because of cloud cover difference but also because of season duration differences. This could have an impact on agronomic performance, and normalizing by the cumulated radiation is a standard practice for comparing agronomic trials (Sadras et al., 2015). Therefore, a first normalization of the data was done by dividing, for each trial, each replication-wise value (for a given genotype and variable) by the cumulated radiation received during the season. The data were then expressed in their usual unit per MJoule (MJ^−1^). The software R (R: A language and environment for statistical computing, R Foundation for Statistical Computing, Vienna, Austria. URL https://www.R-project.org/) was used to perform the statistical analysis.

## Results

### Weather

**Figure 1:**
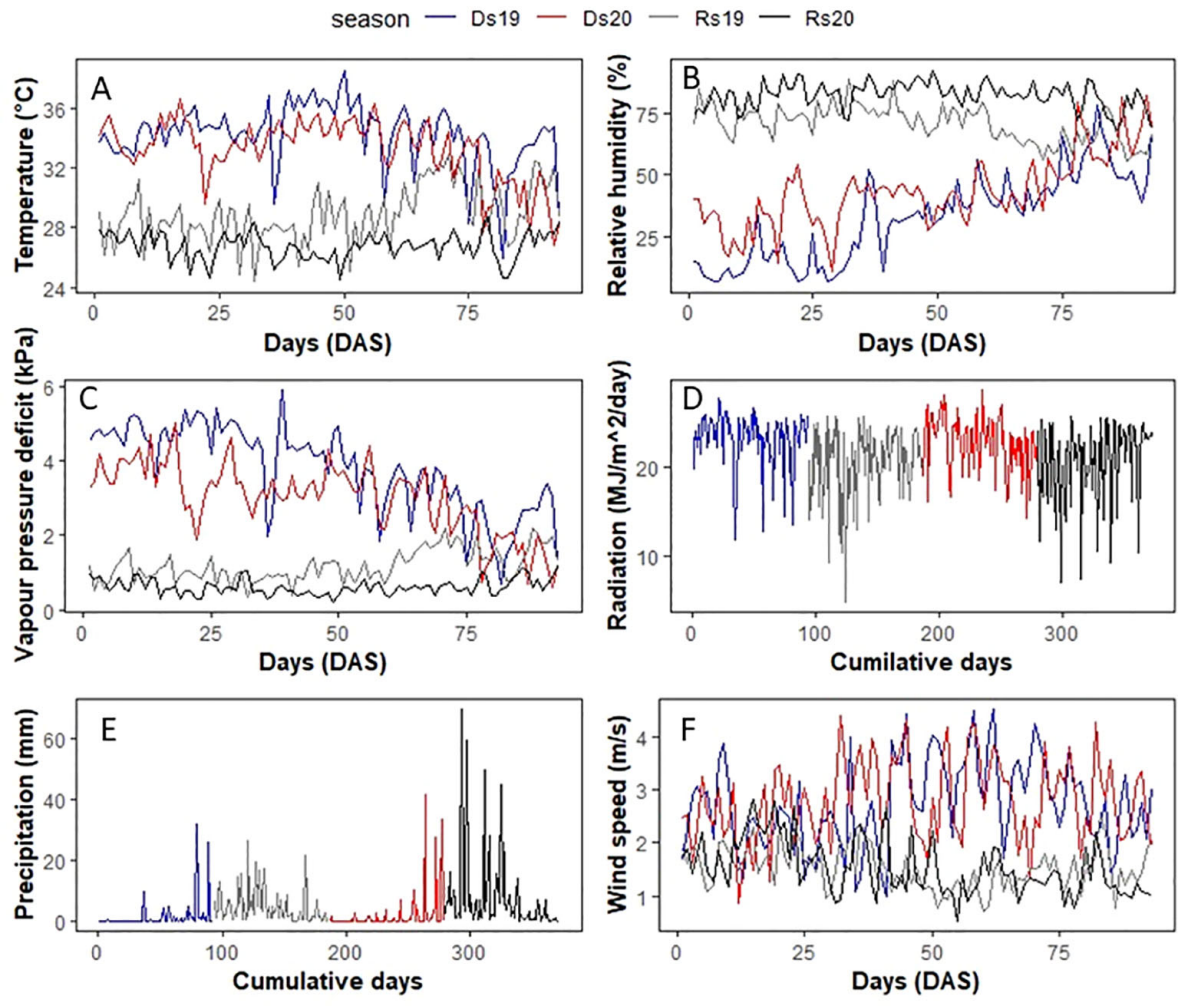
Climatic parameter evolution during the four trials: **(A)** temperature, **(B)** relative humidity, **(C)** VPD, **(D)** radiation, **(E)** precipitation, and **(F)** wind speed. Temperature and relative humidity data in 2019 were collected from a local meteorological station at Sadoré. The 2020 temperature, relative humidity, all radiation data, and wind speed were obtained from NASA. Ds19, dry season 2019; Ds20, dry season 2020; Rs19, rainy season 2019; Rs20, rainy season 2020.

The seasonal measurement of temperature (**Figure 1A**) and RH (**Figure 1B**) showed that the VPD mean values across the four experiments varied between 3.62 kPa (highest-VPD season) and 0.61 kPa (lowest-VPD season, **Table 1**). Dry season trials that were characterized by high temperature and low relative humidity (RH) had high VPD (**Figure 1C**). Ds19 was the hottest and driest season, whose average VPD was 3.62 kPa, whereas Ds20 was a bit cooler/wetter (2.92 kPa) due to an early onset of the rainy season. The rainy seasons had much lower VPD because of rainfall and lower temperature. The mean VPD of Rs19 was 1.14 kPa whereas that of Rs20 was slightly lower (0.61 kPa). Solar radiation was also lower in the two rainy seasons than in the two dry seasons (**Figure 1D**). Precipitations (**Figure 1E**) and wind (**Figure 1F**) varied largely among seasons.

**Table 1:** Seasonal variation of climate factors during the four trials.

Season	Tmean (°C)	RHmean (%)	VPDmean (kPa)	Radcumul (MJ m^2^/season)	Photop (h/day)
Ds19	34.2	32.5	3.6	2,164.5	13
Rs19	28.9	70.8	1.1	1,907.7	13
Ds20	33.2	43.2	2.9	2,135.6	13
Rs20	26.7	82.3	0.6	1,923.4	13

Tmean, temperature mean; RHmean, relative humidity mean; VPDmean, vapor pressor deficit mean; radcumul, cumulative radiation for each trial period; Photop, photoperiod. Ds19 = dry season 2019, Rs19 = rainy season 2019, Ds20 = dry season 2020, and Rs20 = rainy season 2020.

### Differences related to VPD

**Figure 2:**
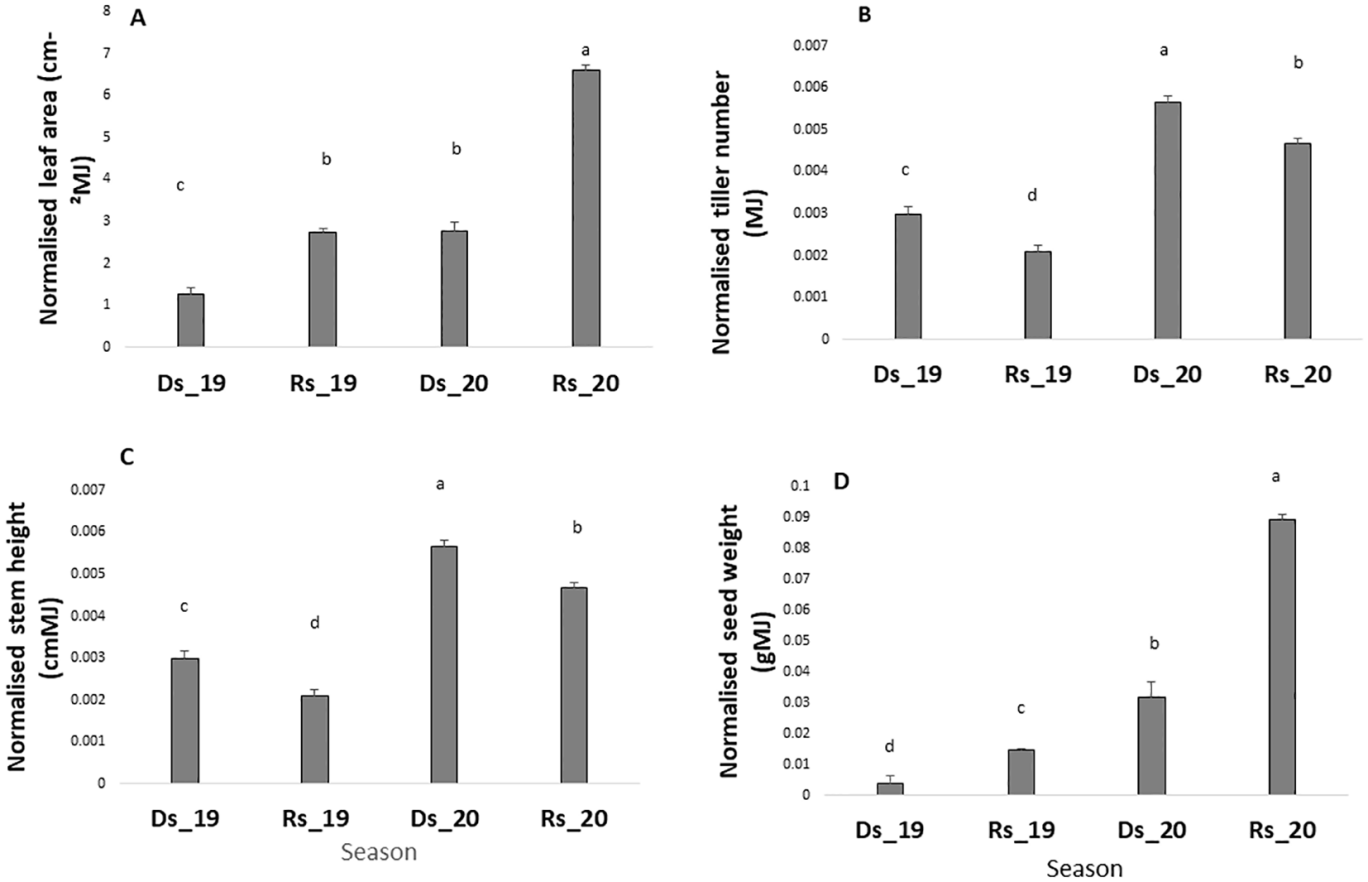
Leaf area **(A)**, tiller number **(B)**, stem height **(C)**, and grain yield **(D)** in the four environments. Values are normalized by the cumulated radiation received in each of the four seasons. Ds19, dry season 2019; Ds20, dry season 2020; Rs19, rainy season 2019; Rs20, rainy season 2020. Bars are means of all genotypes values within each seasons. Bars with same letters do not differ significantly.

After data normalization with radiation, it appeared that the dry season performance was lower than the rainy season one (when comparison for each year was made separately) for key traits such as leaf area and grain yield. As such, the radiation-normalized leaf area was lower in the dry season than in the rainy season (**Figure 2A**) and this in both years. Similarly, the radiation-normalized seed weight was lower in the dry season (Ds19, Ds20) than in the rainy season in both years (**Figure 2D**), whereas stem height and tiller number changed in opposite directions (**Figures 2B, C**).

However, the high performance of the 2020 trials over the 2019 trials suggested a possible field effect on millet performance as trials were conducted in different fields, i.e., one field for the Ds19 and Rs19 trials, and another field for the Ds20 and Rs20 trials. This made the comparison of season effect on trait performance difficult because of that possible field effect. Due to a lack of data on soil chemical composition that could have explained the differences in field performance, we performed a two-way ANOVA with year (2019 and 2020) and season (Ds and Rs) as factors to check for an eventual field effect on data collected across seasons. The year effect represented the field effect, whereas the season effect could be explained by the differences in VPD. The ANOVA showed a highly significant field effect on growth and yield data and also an interaction between the field and year for seed weight (**Table 2**). These interactions indicated that, at least for seed yield, certain germplasm perform better in specific year-season combinations.

**Table 2:** Two-way ANOVA with year and season as factors to check for a related year effect (field) on the measured variables.

Two-way ANOVA	Leaf area	Tiller number	Stem height	Seed weight
**Field effect**	***	***	***	***
**Season effect**	***	***	***	***
**Field × season effect**	**ns**	**ns**	**ns**	**

ns indicates there is no significantly effect. Ds, dry seasons; Rs, rainy seasons.

Significance at *0.05, **0.01, and ***0.001 level. ns, not significant.

To eliminate this field effect on plant performance, and to be able to robustly assess the season effect on plant performance, we then proceeded to a second normalization for each trial, using the data that came out from the first normalization done above against season’s cumulative radiation. This second normalization was based on the grand mean of each of the traits that were measured. These grand means of plant performance for the different traits measured (for example leaf area or yield) represented the overall trait performance in a given field for the whole set of genotypes. Therefore, the second normalization consisted, in a given trial, in dividing replication-wise values (for all genotypes and for a given trait) by the grand mean of the same trait for the considered trial. This second normalization was a way to remove the field effect on that performance, and then to keep only the relative genotypic differences. Therefore, these double-normalized data allowed to compare genotypic performances within trials, and also to test season effects across trials. It was a necessary step to correctly compare the groups of genotypes with regard to the response of growth and agronomic parameters to season effects. The data that were analyzed for the remaining part of the paper were then unit-less data that represented the variation against a grand mean for a given variable.

### Overall difference among germplasm groups

**Figure 3:**
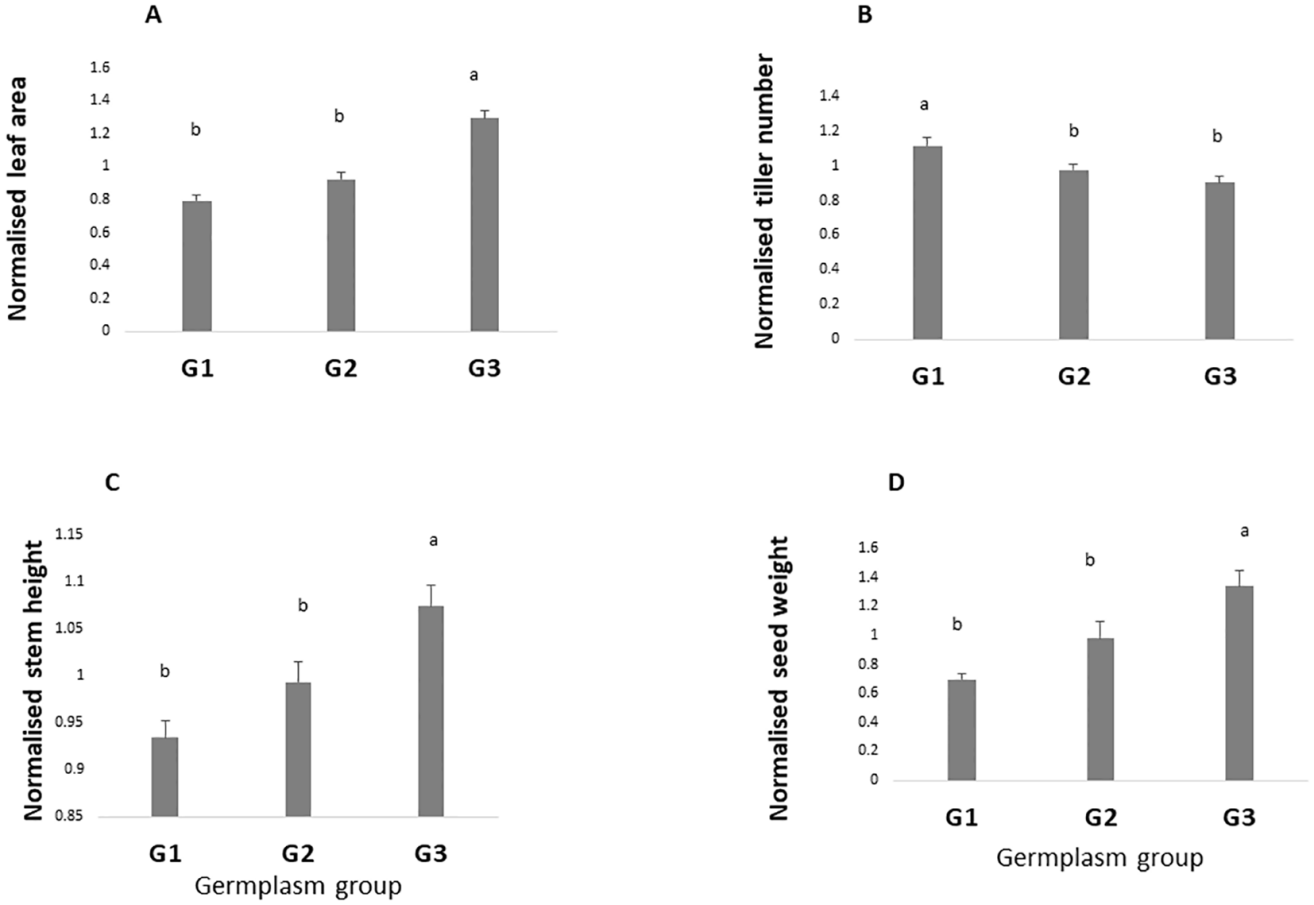
Overall performance of the three-germplasm group. For each trait, values are normalized by the mean. Values are the mean of four seasons (Ds19, Rs19, Ds20, and Rs20). Leaf area **(A)**, tiller number **(B)**, stem height **(C)**, and grain yield **(D)**. Groups with the same letters are not significantly different. G1, G2, and G3, germplasm from humid, semi-humid, and semi-dry regions, respectively; Ds19, dry season 2019; Rs19, rainy season 2019; Ds20, dry season 2020; Rs20, rainy season 2020.

**Figure 3** represents the performance of the three germplasm groups across the four trials, which is without considering any season in particular. Results showed large differences among groups of germplasm. For instance, leaf area was significantly higher in G3 than G2 and G1 (1.29, 0.92, and 0.79, respectively, p value < 0.0001) (**Figure 3A**). Similar results were observed for basal stem height and seed weight (p value <0.0001 and 0.0001, respectively) with G3 showing the highest values (**Figures 3C, D**). By contrast, the high tillering ability was lower in G3 and G2 as compared with G1 (p value < 0.0001) (**Figure 3B**).

### Comparison of the germplasm groups’ performance within each season

**Figure 4:**
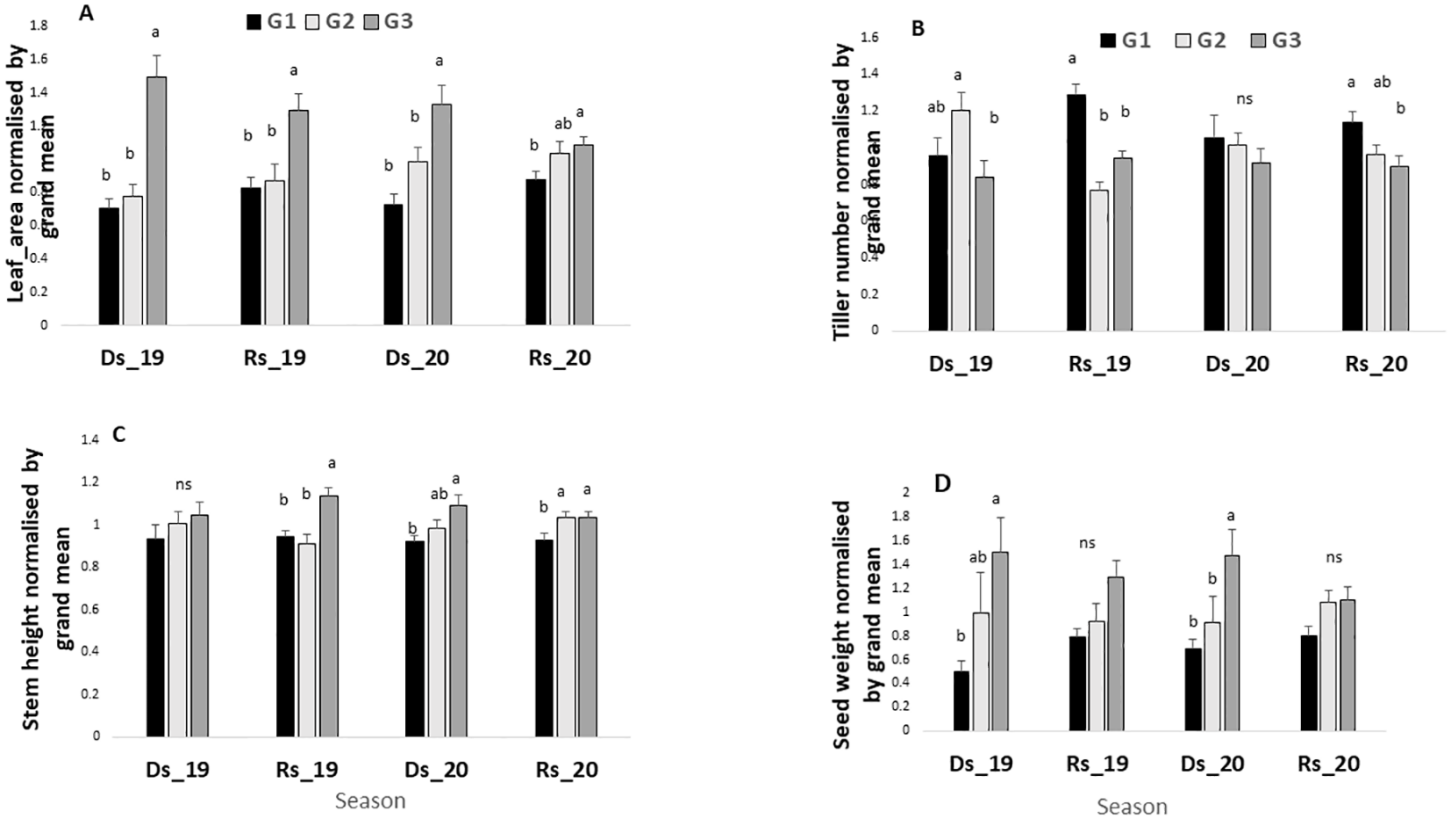
Leaf area **(A)**, tiller number **(B)**, stem height **(C)**, and grain yield **(D)** in the four environments for the three-germplasm group compared between them. For each season, values are normalized by the grand mean. Ds19, dry season 2019; Rs19, rainy season 2019; Ds20, dry season 2020; Rs20, rainy season 2020. G1, G2, and G3 = germplasm from humid, semi-humid, and semi-dry regions respectively. For each season, bars with same letters do not vary significantly.

The ANOVA shown in **Table 2** revealed significant season-by-field interactions, indicating that certain germplasm performed better in specific seasons. Hence, **Figure 4** compares the performances among germplasm groups within each of the seasons. Results indicate that across all seasons (with high and low VPD), leaf area was significantly higher in G3 than in G1 and G2 (**Figure 4A**). By contrast, the tiller number was significantly higher in G1 in the two low VPD seasons (1.14 kPa and 0.61 kPa), and then in G2 in the one high VPD season (3.62 kPa), as compared with G3 (**Figure 4B**). Stem height showed no clear group difference across the different seasons, except for the higher stem height of the G3 group than G1 and G2 in the two low-VPD seasons (1.14 kPa and 0.61 kPa) and one high-VPD season (2.92 kPa) (**Figure 4C**). Notably, while there was no significant yield difference among the three germplasm groups in the two rainy seasons, G3 had a higher grain yield than G1 in both dry seasons, and then G2 in one of the two dry seasons (**Figure 4D**).

### Agronomic performance of each germplasm group across the four VPDs

**Figure 5:**
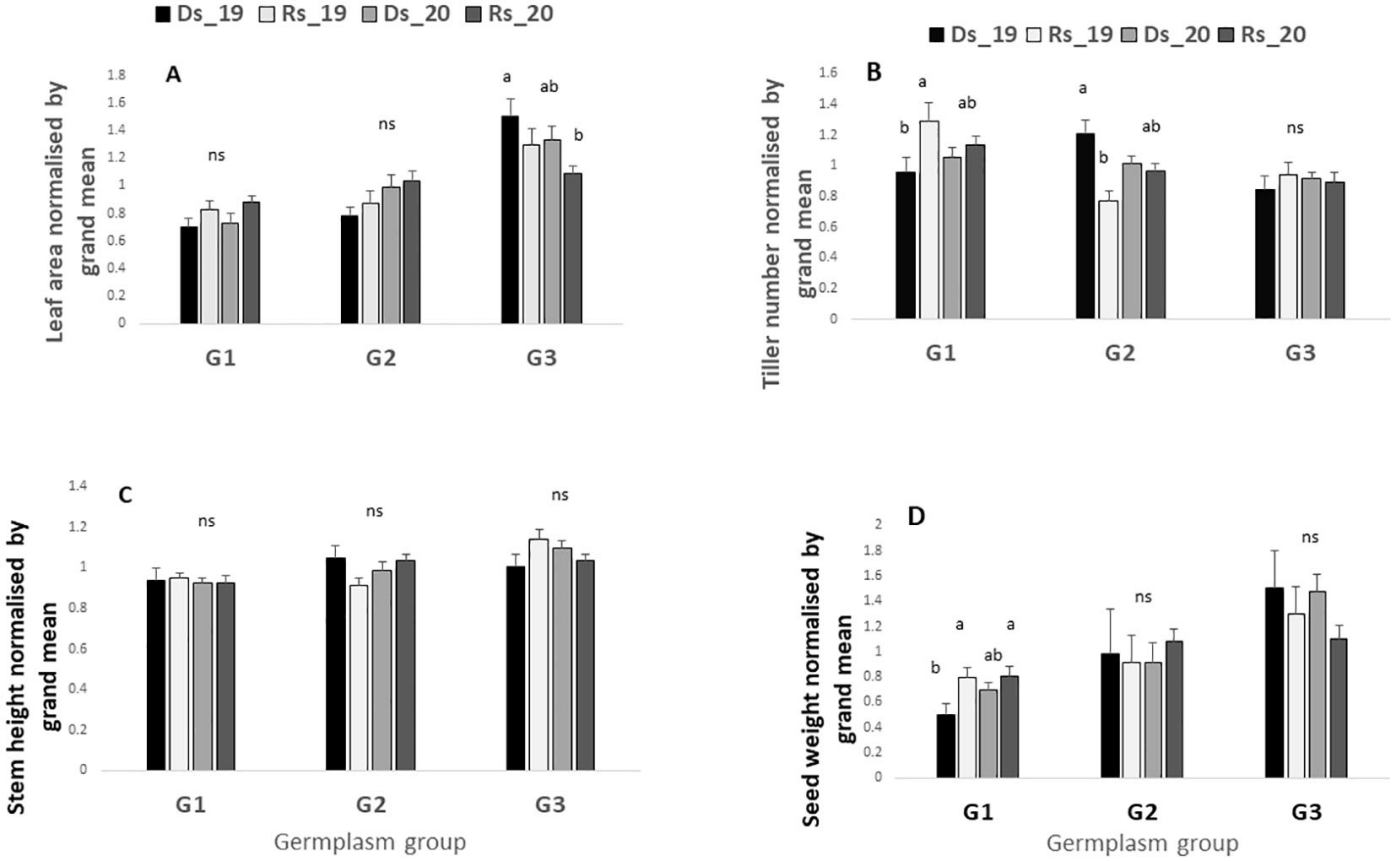
Leaf area **(A)**, tiller number **(B)**, stem height **(C)**, and grain yield **(D)** in the four environments for each germplasm group. For each trait, values are normalized by the grand mean of each season. Ds19, dry season 2019; Rs19, rainy season 2019; Ds20, dry season 2020; Rs20, rainy season 2020; G1, G2, and G3, germplasm from humid, semi-humid, and semi-dry regions, respectively. For each group bars with same letters do not vary significantly.

**Figure 5** represents a comparison within the germplasm group of their performance across the four seasons. **Figure 5A** shows that across the four environments (Ds19, Rs19, Ds20, Rs20), only G3 showed a variability for leaf area with a significant decrease in Rs20, which was one of the two low-VPD environments. By contrast, even though the leaf areas for G1 and G2 seem to respond more favorably to a VPD decrease, this difference was not statistically significant (**Figure 5A**). Results revealed that when the VPD is above (2.9 kPa), the tillering ability of G1 decreased whereas the environmental change seemed to have no effect on G3 tiller number (**Figure 5B**). Unexpectedly, a high tiller number was observed for G2 under high VPD in the dry season (Ds19) (**Figure 5B**). Results revealed also that under high-VPD conditions, the seed weight was reduced in G1 whereas it tended to increase in G3, although the statistical test showed that this increase was not significant (**Figure 5B**).

**Figure 6:**
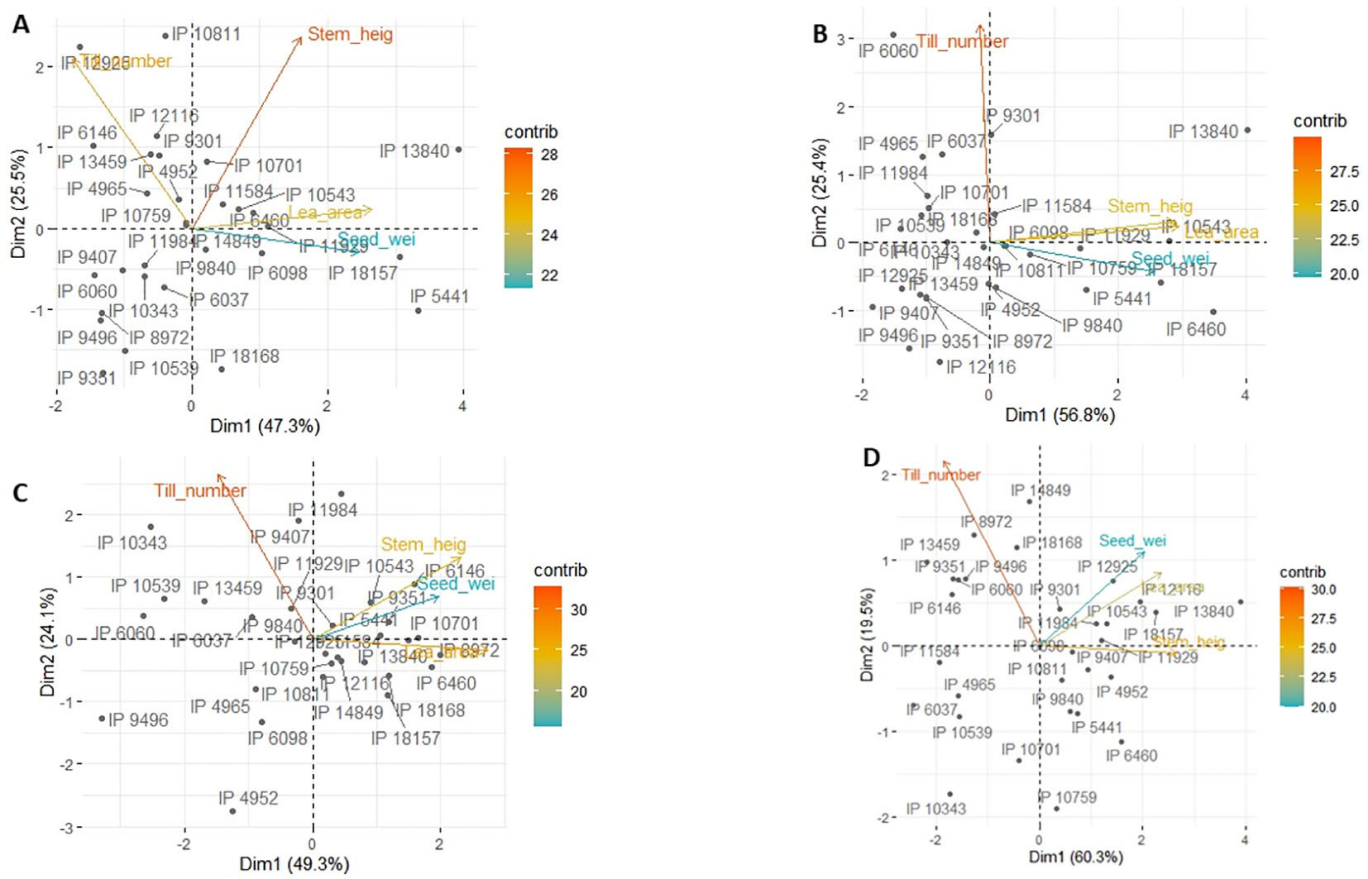
Principal component analysis showing the 30-germplasm performance and the contribution of each trait to each of the principal components for Ds19 **(A)**, Rs19 **(B)**, Ds20 **(C)**, and RS20 **(D)**.

**Figure 6** is a principal component analysis for each of the four trials. The first two vectors explained 75%–80% of the variation. Clearly, the first vector highlighted high yields and came with high positive loading of leaf area and to some extent stem height. By contrast, tiller number had no weight on this vector. Germplasm having high loading on that vector were mostly group 3 germplasm (**Table 3**), especially in the trials taking place in dry years (**Figures 6A, C**), for instance IP13840, IP5441, IP6460, IP10543, or IP18168.

**Table 3:** List of germplasm used in the experiments.

Group 1	Group 2	Group 3
IP 4965	IP 11984	IP 6098
IP 8972	IP 18157	IP 18168
IP 6037	IP 6146	IP 10701
IP 9351	IP 10811	IP 10543
IP 10343	IP 12116	IP 13459
IP 4952	IP 10759	IP 9840
IP 14849	IP 11584	IP 5441
IP 9301	IP 9496	IP 13840
IP 11929	IP 9407	IP 10539
IP 6060	IP 12925	IP 6460

## Discussion

### Season differences

The phenotypic variation observed in crop yield includes genetic and environmental and the interaction between them (Hamidou et al., 2013), although in many cases, the largest sources of variation come from the environment (Sadras et al., 2015). The 2019 and 2020 trials were conducted on different fields inside the research station. While no information on the chemical and mineral composition of these soils was available—it was assumed that the two fields had similar fertility levels. However, the two-way ANOVA with year (2019 and 2020) and season (Ds and Rs) as factors showed that there was a field effect on the measured variates (**Table 2**). In similar trials which are conducted on different fields and in several seasons, classifying varieties according to yield difference (or any measured variate) is likely to be biased (Sadras et al., 2015). This is why it was important to normalize the data in order to separate the part of variation related to fields and that due to VPD. In addition, another normalization was done by dividing the first normalization data by the grand mean for each of the variables. The normalization by the cumulated radiation then by the grand mean of each trial allowed to compare crop performance as affected by other factors such as VPD, independently from the field and the radiation effects.

In pearl millet, both temperature and photoperiod are known to influence growth (Begg and Burton, 1971; Sanon et al., 2014; Vadez et al., 2012b). In our experiments, the average temperatures (34.29°C, 33.29°C, 28.9°C, and 26.76°C, respectively, for Ds19, Ds20, Rs19, and Rs20) were within the limit of good growth of millet (Ong and Monteith, 1985) as well as radiation (Singh et al., 1998). Rain was not deficient during the two rainy seasons (Marteau et al., 2011), and the trials were conducted without any water deficit in the soil. **Table 1** shows that the two dry seasons differed very little in terms of average temperature (34.29°C and 33.29°C) and radiation (23.27 MJ m^−2^ day^−1^ and 22.96 MJ m^−2^ day^−1^), so did the two rainy seasons 28.9°C 26.76°C and 20.51 MJ m^−2^ day^−1^ and 20.68 MJ m^−2^ day^−1^ for mean temperature and radiation, respectively. The photoperiod was almost the same for all seasons (13 h). Hence, most differences came from VPD and genotypes.

After first normalization, growth and yield were higher in the season with the lowest VPD, indicating a positive effect of the low-VPD condition on both growth and yield in pearl millet. Several reports show a positive effect of low evaporative demand on growth (Darlington et al., 1997; Devi et al., 2015; Leuschner, 2002; Yuan et al., 2019). Leaf expansion of maize leaf number 6 is sensitive to high VPD, and there is genetic variation for that trait (Reymond et al., 2003; Welcker et al., 2006). Here, our measurements were coarser since they were done in the field in a destructive manner. Yet, they were able to pinpoint a VPD effect on the leaf area development after normalizing field and radiation differences.

### Germplasm group difference

High evaporative demand has an impact on both growth and yield process depending on the germplasm group. The G1 group showed a decrease in leaf area and seed weight under high VPD. This could be explained by the leaf expansion sensitivity to high VPD which decreased leaf area and by the same occasion radiation interception then yield. In addition to less radiation capture, the yield decline in G1 group could also be temperature-related, since the reproduction period is sensitive to high temperature, and high VPD and high temperature often occur together (Hamidou et al., 2013). **Figures 1A, C** indeed show that high temperature occurred during a period when most entries came to flowering (between 40 and 50 DAS, data not showed). So, the drastic yield decrease during high-VPD season may be due to reproductive sterility and/or flower abortion. These results are similar to previous works, which reported that high VPD had a negative effect on seed set at higher temperature in crops including pearl millet (Hamidou et al., 2013; Gupta et al., 2015). G2 did not show any clear response to VPD but was slightly less tolerant to high VPD as compared with G3.

In this work, tolerance to high-VPD conditions was akin to sustaining leaf area and stem height under high VPD conditions, as this was related to higher yield (**Figure 6**), which also validated our second hypothesis. From this standpoint, the G3 group would be the most tolerant one as it showed the highest yield (biomass and grain) under the highest-VPD seasons (**Figure 4D**) as compared with G1 which decreased the same traits during the same seasons. These results are similar to previous works on water stress (Blum and Sullivan, 1986). These authors reported that drought resistance, measured as the degree of growth inhibition under stress, was higher in races from dry regions than in races from humid regions in a landrace comparison of sorghum and pearl millet from dry and humid regions. The decrease of leaf area in G1 and G2 is a morphological adaptation that enables them to withstand high-VPD periods. Our results differed from Zhang et al. (2005) who compared a population of *Populus divana* to different soil water deficit intensities. They showed that water stress affected dry matter accumulation and allocation more in the dry climate ecotype than in the wet climate ecotype. Although the two types of droughts, atmospheric drought in our experimentation and soil drought in Zhang et al.’s study, are different, the consequences on plant were reported to be similar (Sulman et al., 2016).

## Conclusion

VPD had a major effect on growth and yield of pearl millet. We observed a decrease in leaf area and grain yield not related to soil water status (since enough water was supplied to the crop), but this decrease did not affect germplasm originating from dry areas, or it affected it less. These results are very important to consider when breeding for pearl millet performance for future climate and highlight the potential of Sahelian germplasm to be used as a donor of climate-resilient alleles for the breeding of varieties for areas facing high-VPD conditions and for future climate.

## Data Availability

The raw data supporting the conclusions of this article will be made available by the authors, without undue reservation.
